# The threatening but unpredictable *Sarcoptes scabiei*: first deadly outbreak in the Himalayan lynx, *Lynx lynx isabellinus*, from Pakistan

**DOI:** 10.1186/s13071-016-1685-0

**Published:** 2016-07-19

**Authors:** Khalid Hameed, Samer Angelone-Alasaad, Jaffar Ud Din, Muhammad Ali Nawaz, Luca Rossi

**Affiliations:** Department of Zoology, Mirpur University of Science & Technology (MUST), Mirpur Azad Jammu & Kashmir, Pakistan; Department of Zoology, Arid Agriculture University, Rawalpindi, Pakistan; Institute of Evolutionary Biology and Environmental Studies (IEU), University of Zürich, Winterthurerstrasse 190, 8057 Zürich, Switzerland; Estación Biológica de Doñana, Consejo Superior de Investigaciones Científicas (CSIC), Avda, Américo Vespucio s/n, 41092 Sevilla, Spain; Snow Leopard Foundation, Islamabad, Pakistan; Institute of Biological Sciences, Faculty of Science, University of Malaya, 50603 Kuala Lumpur, Malaysia; Department of Animal Sciences, Quaid-I-Azam University, Islamabad, Pakistan; Dipartimento di Scienze Veterinarie, Università degli Studi di Torino, Via Leonardo da Vinci 44, I-10095 Grugliasco, Italy

**Keywords:** *Sarcoptes scabiei*, *Lynx lynx isabellinus*, Human-lynx conflict, Chitral District, Pakistan, Neglected parasite, Emerging disease

## Abstract

Although neglected, the mite *Sarcoptes scabiei* is an unpredictable emerging parasite, threatening human and animal health globally. In this paper we report the first fatal outbreak of sarcoptic mange in the endangered Himalayan lynx (*Lynx lynx isabellinus*) from Pakistan. A 10-year-old male Himalayan lynx was found in a miserable condition with severe crusted lesions in Chitral District, and immediately died. Post-mortem examination determined high *S. scabiei* density (1309 mites/cm^2^ skin). It is most probably a genuine emergence, resulting from a new incidence due to the host-taxon derived or prey-to-predator cross-infestation hypotheses, and less probable to be apparent emergence resulting from increased infection in the Himalayan lynx population. This is an alarming situation for the conservation of this already threatened population, which demands surveillance for early detection and eventually rescue and treatment of the affected Himalayan lynx.

## Letter to the editor

Although affecting more than 100 species of mammals worldwide [1, 2], the epidemiology of *Sarcoptes scabiei* is still not well understood, with differences between locations and host species [3]. The emerging of *S. scabiei* is frightening, since it may entail devastating mortality in wild and domestic animals, even only from the introduction of a single case [4, 5]. Sudden outbreaks of *S. scabiei* in human, wild and domestic populations have frequently been reported [6]; nevertheless, there is no report of *S. scabiei* infestations in the Turkestan subspecies of the Eurasian lynx, also named Himalayan lynx (*Lynx lynx isabellinus*).

**Fig. 1:**
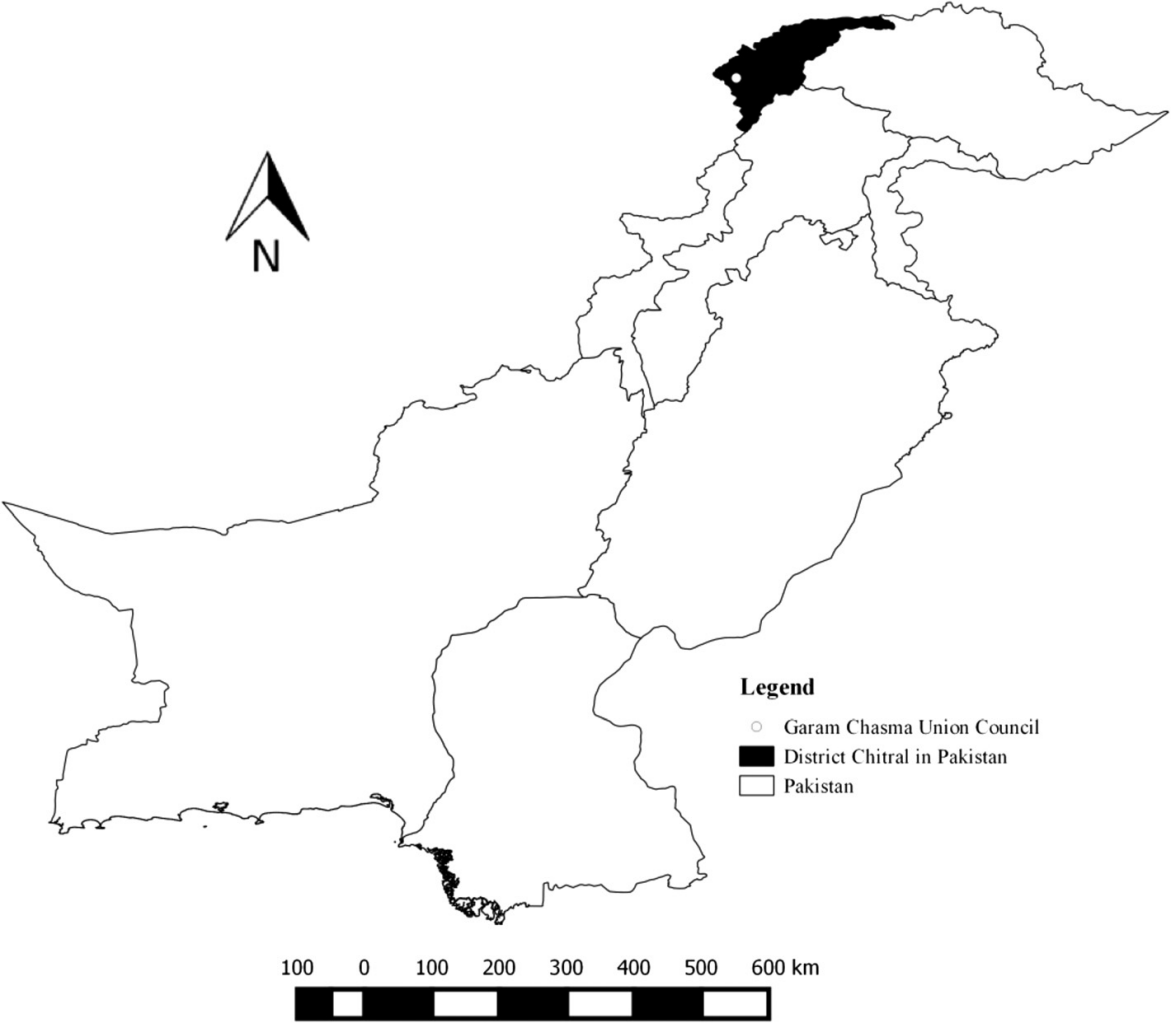
Map of Pakistan showing the site where the mange-infested Himalayan lynx was found

The Himalayan lynx in the Hindu Kush mountain range of the District Chitral, Pakistan (Fig. 1), is highly threatened. The last population assessment reported sporadic occurrence with a minimum of six individuals [7]. The prime threats to the existence of the Himalayan lynx are retaliatory killing because of human-lynx conflict, loss of natural prey-base and loss of habitat to a lesser extent [8].

**Fig. 2:**
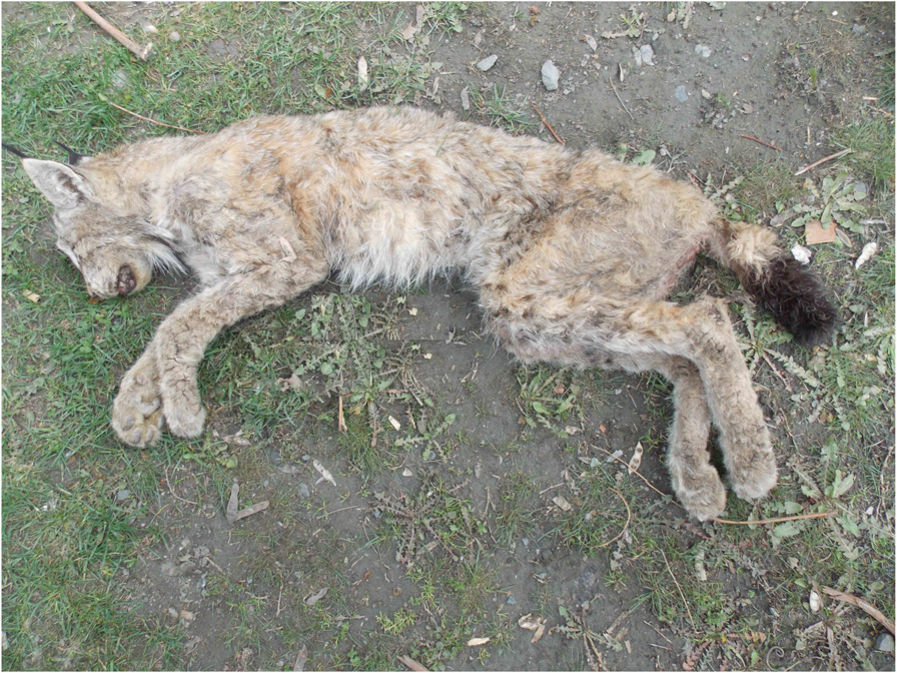
Carcass of the mange-infested Himalayan lynx showing severe crusted lesions

**Fig. 3:**
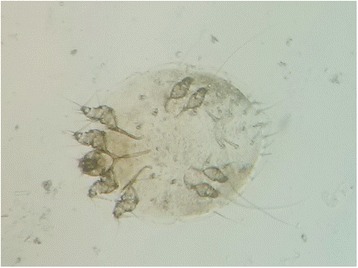
Adult *Sarcoptes* mite taken from the skin scraping of the dead Himalayan lynx

On the 26th of March 2016, a 10-year-old male Himalayan lynx was found by villagers of Karimabad, while in a miserable condition, with severe crusted lesions on the lower limbs (Fig. 2). Although immediately transported by field staff of the Snow Leopard Foundation, to the Animal Hospital in Chitral City, the lynx died before treatment was started. Deep skin scrapings were collected and examined following KOH clearing [9] (Fig. 3).

This is the first report of fatal outbreak of sarcoptic mange in the Himalayan lynx (*Lynx lynx isabellinus*) from Pakistan. A high *Sarcoptes* mite density was detected (1309 mites/cm^2^ skin), with prevailing larval stages. We considered two hypotheses, which could explain the origin of the outbreak.

Hypothesis (i): The outbreak is ‘genuine’ emergence of an infestation, which is new to the Himalayan lynx population. Likely sources could be other carnivores sharing habitat with the Himalayan lynx, such as wolf, snow leopard, jackal, fox and leopard cat, according to the host-taxon derived hypothesis [10]. While in nearby Central Karakhoram National Park, Gilgit-Baltistan, one of the Authors (LR, unpublished) collected photo trap evidence that scabies was present among red foxes (*Vulpes vulpes*). In western Mongolia numerous reports have been made of a debilitating mange-like affliction in the snow leopard (*Uncia uncia*); however no skin samples have been collected [11]. Similarly, a mange-like condition was observed (though not laboratory confirmed) in a snow leopard captured near Skardu, Gilgit-Baltistan [12]. In Scandinavia and Switzerland, deadly sarcoptic mange in Eurasian lynx has been associated to epidemic or endemic disease in the sympatric abundant red fox populations [13]. Other putative sources are infested domestic animals, through prey-to-predator cross-infestation [14]. Livestock, especially lambs and kids, are major victims of lynx attacks [8]. Most households of the community hold small herds composed of one or two cattle and ten to fifteen sheep and goats. During summer domestic animals are taken to alpine pastures for grazing, and are more vulnerable to predation by lynx. Sarcoptic mange is widespread amongst small domestic ruminants in Pakistan [15].

Hypothesis (ii): It is ‘apparent’ emergence/re-emergence, where *Sarcoptes* infestation was pre-existing, and the new recognition is a result of increased detection opportunities [7].

This is the first *Sarcoptes* mite infection case report in the Himalayan lynx population from Pakistan. This is an alarming situation for the conservation of the already threatened population of this species, which demands surveillance for early detection and eventually rescue and treatment of the affected animals.

## Abbreviations

Not applicable.
